# Managerial leadership for research use in nursing and allied health care professions: a narrative synthesis protocol

**DOI:** 10.1186/2046-4053-3-57

**Published:** 2014-06-05

**Authors:** Wendy A Gifford, Paul Holyoke, Janet E Squires, Douglas Angus, Lucie Brosseau, Mary Egan, Ian D Graham, Carol Miller, Lars Wallin

**Affiliations:** 1 The University of Ottawa, 451 Smyth Road, K1H 8M5 Ottawa, ON, Canada; 2 Saint Elizabeth Health Care, 90 Allstate Parkway, Suite 300, L3R 6H3 Markham, ON, Canada; 3 Ottawa Hospital Research Institute, 451 Smyth Road, K1H 8 M5 Ottawa, ON, Canada; 4 Canadian Physiotherapy Association, 955 rue Green Valley Crescent, Suite 270, K2C 3 V4 Ottawa, ON, Canada; 5 Dalarna University, Högskolan Dalarna, 791 88 Falun, Sweden

**Keywords:** Dietetics,implementation science, Knowledge translation, Leadership, Management, Nursing, Occupational therapy, Physiotherapy, Research use, Speech and language pathology

## Abstract

**Background:**

Nurses and allied health care professionals (physiotherapists, occupational therapists, speech and language pathologists, dietitians) form more than half of the clinical health care workforce and play a central role in health service delivery. There is a potential to improve the quality of health care if these professionals routinely use research evidence to guide their clinical practice. However, the use of research evidence remains unpredictable and inconsistent. Leadership is consistently described in implementation research as critical to enhancing research use by health care professionals. However, this important literature has not yet been synthesized and there is a lack of clarity on what constitutes effective leadership for research use, or what kinds of intervention effectively develop leadership for the purpose of enabling and enhancing research use in clinical practice. We propose to synthesize the evidence on leadership behaviours amongst front line and senior managers that are associated with research evidence by nurses and allied health care professionals, and then determine the effectiveness of interventions that promote these behaviours.

**Methods/Design:**

Using an integrated knowledge translation approach that supports a partnership between researchers and knowledge users throughout the research process, we will follow principles of knowledge synthesis using a systematic method to synthesize different types of evidence involving: searching the literature, study selection, data extraction and quality assessment, and analysis. A narrative synthesis will be conducted to explore relationships within and across studies and meta-analysis will be performed if sufficient homogeneity exists across studies employing experimental randomized control trial designs.

**Discussion:**

With the engagement of knowledge users in leadership and practice, we will synthesize the research from a broad range of disciplines to understand the key elements of leadership that supports and enables research use by health care practitioners, and how to develop leadership for the purpose of enhancing research use in clinical practice.

**Trial registration:**

PROSPERO CRD42014007660.

## Background

Closing the gap between what is ‘known’ from high-quality research evidence and what is ‘done’ in clinical practice (the research-practice gap) is a national and international imperative to ensure optimal health outcomes for patients and provide more effective health care services [1,2]. The use of research evidence by health care clinicians will help close the research-practice gap; however, research use in clinical practice remains unpredictable and inconsistent across all health care disciplines [3-5]. Widely cited reports estimate that 30% to 45% of patients do not receive proven, effective treatments while 20% to 25% of care is unnecessary or potentially harmful [6,7]. Together, nursing, physiotherapy, occupational therapy, speech and language pathology, and dietetics typically form over half the clinical health care interprofessional workforce [8]. The leadership of front line and senior level managers is consistently described in implementation science research as a critical factor to enabling research use by nurses and other health care professionals [9-18]. However, this important literature has not yet been synthesized and a lack of clarity exists about what aspects of leadership are empirically related to research use. Identifying these aspects of leadership is fundamental to designing effective interventions and guiding creation of sound organizational strategies to improve the delivery of high-quality evidence-based health care and ultimately improve patient, provider and system outcomes.

Although the value of leadership is well recognized in health care, what leaders actually do (that is, their behaviours) to enable, support and sustain research use effectively for quality patient care and positive patient outcomes is not well understood. Research on barriers to research use in health care consistently identifies the behaviours of managers and their lack of leadership as major limiting factors to research use by clinicians [19-23]. This is not surprising, as the work of managers and clinicians in nursing and allied health is highly interrelated and generally defined through policies, procedures and infrastructures developed and maintained by management [20]. Practice change interventions targeting front line staff in nursing, physiotherapy, occupational therapy, speech and language pathology and dietetics typically require change in individual practice as well as change in institutional practice. Thus, interventions that target leadership of managers can have far-reaching effectiveness in these professions, as they target people with the positional power to make necessary institutional and infrastructure changes to support and sustain changes that enable research use in clinical practice.

A literature review of quality improvement research in health care has shown evidence to support an association between leadership and the successful implementation of practice change related to quality improvement and patient outcomes [22]. The review showed that the actions and inactions of managers were positively and negatively associated with implementing quality improvement initiatives, wherein certain types of leadership behaviours were associated with successful quality improvement, and other types were associated with failures. For example, the failure of senior leaders to provide resources and support for clinical audits contributed to patients being harmed and lives lost in the UK Bristol tragedy [24], while passive senior and clinical leadership support was attributed to a failure in changing physicians’ and nurses’ practices for depression care in nine primary health care clinics in the United States [25]. Similarly, positive quality improvement changes were associated with managers’ commitment and understanding of quality improvement principles, their close working relations with staff, and perceptions of them as ‘open minded and communicative’ [26]. However, the quality improvement literature does not delineate leadership behaviours for enabling nursing and allied health care professionals’ use of research evidence in clinical practice, and the management support for quality improvement efforts might be different from that required for implementing research-based change.

While the context and setting are recognized as having a powerful influence on research use [12,27,28], they can also affect the impact of leadership [29-32]. A large international cross-cultural study of leadership in business found that some leadership practices (such as value-based participative leadership behaviours) are effective across different contexts, while others (such as autonomous behaviours) are contextually and culturally specific [33]. Different health care settings (for example, acute care in hospitals or community care) have different contextual factors that influence research use, and health care disciplines differ in training, education, knowledge and scope of practice. Thus, effective leadership in one setting or professional group might not seamlessly transfer to another.

With nurses, physiotherapists, occupational therapists, speech and language pathologists and dietitians comprising the largest proportion of the interprofessional health care team, maximizing their research use has the potential to substantially improve the process of health care delivery.

The overall purpose of this systematic review is to synthesize the evidence on front line and senior managers’ leadership behaviours associated with the use of research evidence by nurses and allied health care professionals (physiotherapists, occupational therapists, speech and language pathologists and dietitians), and determine the effectiveness of interventions that promote these behaviours amongst health care leaders. Specific objectives are: (1) to identify the frontline and senior managers’ leadership behaviours associated with research use by clinical staff in these professions; (2) for behaviours identified in Objective 1, to determine the effectiveness of interventions for developing such leadership behaviours.

Since the effectiveness of leadership might not be similar across different settings, we will synthesize the literature within each professional group by setting (acute, community and long-term care), and then synthesize findings across all professional groups and settings. The knowledge generated from this review will help unpack the ‘black box’ of leadership and advance the science of leadership as a knowledge translation strategy to improve health care quality and patient outcomes.

This protocol has been registered with PROSPERO, no CRD42014007660.

## Methods/Design

A narrative synthesis will be conducted to synthesize diverse forms of evidence. Narrative synthesis integrates evidence from multiple research designs, including experimental, quasi-experimental and observational studies to generate new insights for policy and practice and will be helpful in generating hypotheses for future experimental studies [34]. This approach has been selected because the majority of leadership and management research is descriptive [14,35]. A review of leadership research in health and business between 1970 and 1999 found that only 15 of 6,628 articles (0.2%) investigated a correlation between leadership and measureable outcomes [35]. With recent interests in health care leadership and research use, however, we anticipate more studies.

We will use a systematic four-stage approach to conduct the review, in line with methodological guidelines set forth by Grimshaw [36].

### Searching the literature

Shaped by the review objectives, the search strategy will be refined as the review proceeds and the parameters are further tested [34]. The search strategy will use a mix of controlled terminology (MeSH or Cumulative Index to Nursing and Allied Health Literature headings) and keywords to ensure adequate subject coverage, based on the concepts of leadership, management, research use, implementation, knowledge translation (transfer) and health disciplines. Limits will be applied for language (French and English only). No limits will be applied for date of publication.

The following electronic bibliographic databases will be searched: Medline (Ovid), Cumulative Index to Nursing and Allied Health Literature, Cochrane Database of Systematic Reviews, Cochrane Central Controlled Trials Register, Database of Abstracts of Reviews of Effects, Proquest Nursing and Allied Health, Proquest Dissertations and Theses, ABI Inform Global, EMBASE, Psycinfo, SCOPUS and PEDro.

To detect additional eligible references, we will also cross-check reference lists from articles identified in the preceding search and conduct focused hand searching of selected journals known in the field for the last five years (for example, *Academy of Management Journal*, *Implementation Science*). Additional information will be sought from authors of identified references as required. We will also search key websites for grey literature, for example the Canadian Health Leadership Network [37].

### Study eligibility criteria

Reflecting a ‘PICO’ (‘participants, intervention, comparator, outcomes’) approach, the following criteria will guide study selection for each objective.

#### Objective 1

To identify leadership behaviours associated with research use.

##### Study design

Studies reporting on original research using experimental, quasi-experimental and observational designs (for example, case study, before-and-after studies, observational, qualitative studies).

##### Participants

Studies must include nurses, physiotherapists, occupational therapists, speech and language pathologists, or dietitians.

##### Interventions

Studies with interventions involving front line or senior level managers for the purpose of influencing clinical staff to use research will be included, using the Cochrane Effective Practice and Organization of Care taxonomy for organizational interventions [38]. Interventions may include (but are not limited to): revisions to managers’ professional roles, changes to the organization of management team structures, integration of management services with other services, or changes to the organization of managers’ practices.

##### Comparator

For studies using an experimental design, we will compare interventions involving managers for the purpose of influencing research use in clinical staff with interventions that do not involve managers, or with no intervention.

##### Outcomes

Quantitative studies must include quantitative outcome measures of research use at the practitioner level (for example, change in clinical practice), patient level (for example, change in health outcome), or health care resource utilization level (for example, frequency and length of hospital stay, readmission rates). Qualitative studies must specifically focus on manager’s behaviours or activities that facilitate, enable and influence research use by staff.

#### Objective 2

To determine effectiveness of interventions for developing leadership behaviours identified in Objective 1. The same inclusion criteria will apply with the following exceptions.

##### Study design

Based on recommendations from the Cochrane Effective Practice and Organization of Care [38], the following designs will be included: randomized control trials, non-randomized controlled trials, controlled before-and-after studies, and interrupted time series and repeated measures studies.

##### Outcomes

Studies must include an objective measure of one or more of the leadership behaviours identified in Objective 1.

Studies will be excluded if they do not meet the aforementioned inclusion criteria. To avoid duplicating previous systematic reviews [39,40], studies of interventions involving informal opinion leaders or those where opinion leaders were identified as managers will be excluded, as will studies in which the outcomes are the managers’ use of research.

### Study selection

To screen studies for inclusion in the review, a two-step process will be used. First, titles and abstracts identified in the search strategy will be screened independently for inclusion criteria by two team members. Each article will be rated as ‘include’ , ‘exclude’ or ‘unclear’ , meaning not enough information. Full text copies of all citations identified as ‘include’ or ‘unclear’ will be retrieved for formal review. Second, two reviewers will independently assess each study using a standard form developed for this study that outlines the predetermined inclusion criteria. Disagreements throughout this process will be resolved by consensus or third-party adjudication where consensus cannot be reached.

‘Research use’ describes the process of putting knowledge that has been derived from research evidence into action [41]. It is a form of knowledge translation, described as the ‘ethically sound application of knowledge’ [42] to improve the health of populations, whereby the knowledge has a research base to substantiate it.

‘Managers’ are classified by role at two hierarchical levels: front line and senior levels [43]. Front line managers oversee operations and staff or direct patient care, and are typically referred to as managers, unit-level managers, supervisors or coordinators. Senior managers have executive positions that involve wider organizational or departmental operational activities for health care delivery, and have such titles as chief executive, vice president, director, senior executive, executive manager or operating officer [44].

### Data extraction

Study data will be extracted using standard forms and entered into an Excel spreadsheet. Data will be extracted by one reviewer and checked for accuracy and completeness by a second reviewer. Any discrepancies in data extraction will be resolved through discussion re-evaluation of the studies and adjudication with a third senior reviewer. Data will be extracted on, but not limited to: study design, objectives, sample and subject characteristics, settings, theoretical framework, instruments used (including reliability and validity), the intervention and its components, and key findings with respect to outcomes. The data extraction form will be piloted on five studies to refine the form and ensure it captures all of the intricacies of both qualitative and quantitative designs.

### Methodological quality

Methodological quality of studies will be assessed using one of three tools according to study design. Two reviewers will independently assess the quality of included studies; discrepancies will be resolved through discussion or third-party adjudication. Experimental controlled trials will be evaluated using the Cochrane Collaboration risk of bias tool, developed to assess risk of bias critically in five domains: selection bias, performance bias, detection bias, attrition bias, reporting bias and other bias [45]. For each domain, a judgment for risk will be assigned as either ‘low risk’ , ‘high risk, or ‘unclear risk’ (indicating lack of information or uncertainty over the potential for bias) [45]. The Cochrane Collaboration risk of bias tool was developed in 2007 by a group of methodologists and review authors because of the unreliability and lack of validity of previously used quality assessment scales and scores, and is recommended by the Cochrane Collaboration [46] for assessing experimental trials [45].

For all other quantitative studies, methodological quality will be assessed using the Effective Public Health Practice Project Quality Assessment Tool for Quantitative Studies [47,48]. Assessment results from the tool will lead to an overall rating of strong, moderate, or weak in eight sections: selection bias, study design, confounders, blinding, data collection methods, withdrawals and dropouts, intervention integrity, and analysis. This tool has been evaluated for content and construct validity [48] and has shown to have acceptable inter-rater agreement for individual domains and excellent inter-rater agreement for the final grade [49].

Informed by recommendations by the Cochrane Collaboration Qualitative Methods Group, qualitative studies will be critically assessed using a tool to assess the quality of reporting, methodological rigour, and conceptual depth and breadth [50]. The *Quality Checklist for Qualitative Studies*[51] will be used, and has been previously used previously in an extensive narrative review by Greenhalgh and colleagues [52]. This tool assesses 11 areas, including the appropriateness of the study design, whether the context was sufficiently described and whether the analysis was systematic and rigorous. Each area will be described and an assessment for each area will be given, as weak, moderate or strong.

### Data analysis and synthesis

The approach for the narrative synthesis is based on methodological guidelines described by Grimshaw [36] and Popay *et al.*[53], and involves: (a) grouping studies into discrete categories; (b) conducting a ‘within-study’ analysis, and (c) conducting a ‘cross-study’ synthesis of leadership behaviours and interventions within each professional group and across all professional groups.

#### Grouping studies into categories

Study data will be grouped into discrete categories based on level of manager (senior and front line); professional group (nursing, occupational therapy, physiotherapy, speech and language pathology, dietetics), sector (acute, community, long-term care), and research design (experimental, non-experimental, qualitative). Data will be analyzed by the objectives of the review into either leadership behaviours associated with research use or leadership interventions.

#### Within-study synthesis

A narrative description of the findings for each objective of the review will be conducted for each study. Evidence tables that include the quality and risk of bias assessment for each study will be created.

#### Cross-study synthesis

Data will be aggregated and analyzed for patterns within each professional group, and an overall synthesis will be conducted across all included studies. This descriptive analysis will allow us to examine patterns in the data related to leadership behaviours and interventions that are associated with research use and related outcomes within and across professional groups to reflect the interprofessional landscape of health care delivery. The cross-study synthesis will include a description of the number of studies, the relationships in the findings, and the range of effects observed [36,53]. The robustness of the synthesis produced will be demonstrated by the systematic and transparent processes, tools and techniques used to conduct the analysis [53].

Although not anticipated, if there is sufficient homogeneity across groups of studies employing experimental randomized control trial designs, a meta-analysis will be performed. For continuous data, we will present analysis as weighted mean differences with 95% confidence intervals; for dichotomous data, we will present data as pooled odds ratios using an inverse variance method with 95% confidence intervals. The overall intervention effects will be calculated using a random effects model [54]. This model assumes that there is a different underlying effect of each study and takes this into consideration, leading to more conservative confidence intervals. For continuous variables we will use the inverse variance method for meta-analysis, while for dichotomous variables we will use the Mantel-Haenszel method. A test for heterogeneity will be conducted to determine the degree of similarity in the studies’ outcomes. We will assess heterogeneity using the *I*^2^ statistic, which describes the proportion of variability in the summary estimate that is due to heterogeneity; *I*^2^ values greater than 50% indicate substantial heterogeneity. Where data allows, we will explore sources of heterogeneity through subgroup analysis for professional group and sector.

## Discussion

The aim of this systematic review is to synthesize the research evidence on the frontline and senior managers’ leadership behaviours that are associated with research use by clinical staff in five health care professions; we will also assess the effectiveness of interventions to develop these behaviours. Using an integrated knowledge translation approach, the proposed synthesis will address a gap in the literature related to leadership and its association with research use in the five professions. The key outcomes will be: (1) a comprehensive review that synthesizes a wide range of evidence into a meaningful summary of leadership behaviours within and across five different professional groups (nursing, occupational therapy, physiotherapy, speech and language pathology, dietetics); (2) a robust organization of the evidence base on interventions to develop leadership behaviours for research use; (3) an understanding of the context that impacts leadership for research use; and (4) a beginning theory and evidence base on the causal mechanisms in which leadership is associated with research use.

Possible limitations of the proposed review relate to the diversity of terminology used for research use in health care, variations in the reporting of interventions, the range of context and settings in which managers in nursing and allied health work, and challenges synthesizing multiple groups. Inclusion of different types of evidence and strategies for ensuring robustness in the methodology should help address these limitations.

Our synthesis within and across the five health care professions will produce results that are relevant to practice, education and policy in a range of different health care environments. Together, these outcomes will provide knowledge users with practical, actionable and evidence-based information to inform the leadership practices of front line and senior managers, programme development, and governance policies and procedures to support leadership for research use in clinical practice. Outcomes will further inform knowledge users and researchers in the design of interventions to develop leadership for enabling research use in clinical practice with the ultimate goal of improving health care delivery and patient outcomes.

## Competing interests

The authors declare no competing interests.

## Authors’ contributions

WG conceived the project, designed the review, and drafted and revised the manuscript. JS and IG were involved in the design and provided feedback for the idea, concept and research design. PH, DA, LB, ME and LW provided content expertise and feedback on the concept, protocol and manuscript. CM provided content expertise. All authors read and provided final approval of the manuscript.

## Authors’ information

WG holds a joint position as Assistant Professor at the University of Ottawa School of Nursing and Associate Researcher at Saint Elizabeth Health Care. Her field of research is leadership, management and knowledge translation in health research areas.

PH is Director of Research and Program Development at Saint Elizabeth and brings expertise in health care policy and the governance and management of health care organizations.

JS is an Assistant Professor in the University of Ottawa School of Nursing and Associate Scientist in the Clinical Epidemiology Program at the Ottawa Hospital Research Institute. She brings expertise in synthesis methodologies, the study of context, and the design and evaluation of theory-based knowledge translation interventions.

DA is Professor in the Telfer School of Management at the University of Ottawa, has long-standing involvement in the Ontario Training Centre for Health Services and Policy Research, and brings expertise in health economics, health policy and health care management.

LB is a rehabilitation epidemiologist and Professor at the School of Rehabilitation Sciences at the University of Ottawa. She holds a University Research Chair in Evidence-based Practice in Rehabilitation and is a member of the musculoskeletal group at Collaboration Cochrane, and provides expertise for synthesis and interpretation of findings related to physiotherapy and other allied health professions, as well as the use of knowledge translation strategies to enhance research use in physiotherapy.

ME is Professor in the School of Rehabilitation Sciences at the University of Ottawa and a scientist at the Bruyere Research Institute. She brings expertise in allied health practices and the use of knowledge translation strategies to enhance research use in occupational therapy.

IDG is Senior Scientist in the Clinical Epidemiology Program of the Ottawa Hospital Research Institute and Professor in Epidemiology and Community Medicine at University of Ottawa. His extensive research focuses on knowledge translation and conducting applied research on strategies to increase implementation of research findings.

CM is the Director of the Canadian Physiotherapy association.

LW is Professor in Nursing, directed at implementation research at Dalarna University in Sweden. He is also the director of research in the regional health care organization Dalarna County Council. His research is focused on implementation and knowledge use in health care organizations.
